# *Agency matters!* Social preferences in the three-person ultimatum game

**DOI:** 10.3389/fnhum.2013.00312

**Published:** 2013-06-27

**Authors:** Johanna Alexopoulos, Daniela M. Pfabigan, Florian Göschl, Herbert Bauer, Florian Ph. S. Fischmeister

**Affiliations:** ^1^Department of Psychoanalysis and Psychotherapy, Medical University of ViennaVienna, Austria; ^2^Social, Cognitive and Affective Neuroscience Unit, Faculty of Psychology, University of ViennaVienna, Austria; ^3^Department of Neurophysiology and Pathophysiology, University Medical Center Hamburg-EppendorfHamburg, Germany; ^4^Study Group Clinical fMRI, Department of Neurology, Medical University of ViennaVienna, Austria

**Keywords:** altruism, spite, social preferences, MFN, ultimatum game

## Abstract

In the present study EEG was recorded simultaneously while two participants were playing the three-person ultimatum game (UG). Both participants received different offers from changing proposers about how to split up a certain amount of money between the three players. One of the participants had no say, whereas the other, the responder, was able to harm the payoff of all other players. The aim of the study was to investigate how the outcomes of the respective other are evaluated by participants who were treated fairly or unfairly themselves and to what extent agency influences concerns for fairness. Analyses were focused on the medial frontal negativity (MFN) as an early index for subjective value assignment. Recipients with veto-power exhibited enhanced, more negative-going, MFN amplitudes following proposals that comprised a low share for both recipients, suggesting that responders favored offers with a fair amount to at least one of the two players. Though, the powerless players cared about the amount assigned to the responder, MFN amplitudes were larger following fair compared to unfair offers assigned to the responder. Similarly, concerns for fairness which determined the amplitude of the MFN, suggested that the powerless players exhibited negative and conversely the responders, positive social preferences.

## Introduction

Comparative processes are essential to assess the emotional meaning assigned to a given situation. Whether we perceive something as pleasant or unpleasant depends on the alternatives and their accessibility (Ben-Ze'ev, 2000). For example, a rewarding stimulus might get devalued in situations associated with feelings of anger or envy. Thus, the nature of emotions elicited by the reception, omission, or termination of reward or punishment depends on what we expect and on what others receive in comparison to oneself (Festinger, 1954; Rolls, 2005). This circumstance becomes apparent when looking at recent findings in the field of neuroeconomics investigating how people evaluate specific situations associated with reward or punishment in relation to significant others using simple experimental games.

One [besides several others, for a review see, Rilling and Sanfey (2011)] commonly used experimental game to study reward related decision processes and the underlying neural substrates in a social context is the ultimatum game (UG; Güth et al., 1982). In its original version a proposer is endowed with a sum of money he/she has to share with a responder. He/She can send any positive amount to the responder, who in turn has the possibility to reject or accept the proposed division of money. If the proposed distribution is accepted by the responder, the money will be allocated accordingly. Otherwise, if rejected by the responder, both receive nothing. The proposer can make only one proposal, all players are anonymous to each other, and the game ends after the responder has made his/her decision. Of course, the aim of each player in this bargaining game is to maximize his/her share of the money. Nevertheless, most responders are willing to abandon their division if it is smaller than 20% of the total amount and proposers offer about 40–50% of the total amount (Güth et al., 1982; Thaler, 1988; Güth and Van Damme, 1998). Though behavior in this game seems to be rather irrational, results are very robust and do not markedly change with the size of the stake (Slonim and Roth, 1998; Cameron, 1999; Munier and Costin, 2002). Even demographic variables, intellectual abilities, and socio-economic status do not modulate behavior in this game (Güth et al., 2007; Nguyen et al., 2011).

There are several regions in the brain that are implicated in the representation of the subjective value of reward and punishment [for reviews see Schultz (2006); Grabenhorst and Rolls (2011)]. One of these, the anterior cingulate cortex (ACC), and in particular its dorsal part, might be of particular importance in the comparative processes discussed above. In comparison with other areas associated with the representation of reward, the ACC integrates various aspects of a decision, e.g., probability, payoff, and effort (Kennerley et al., 2009, 2011). Furthermore, the ACC evaluates not only values of alternatives during choice but also the consequences of choices made. For this, the ACC receives input from different neuronal sources associated with certain qualities of a reward and has strong connections to motor areas (e.g., Vogt et al., 1992). All of these are requirements needed to synthesize these various aspects of a given situation and to adapt preferences in the light of the current goal and the effort that has to be taken. However, this region is not necessarily related to actual decision behavior (Seo and Lee, 2007; Luk and Wallis, 2009).

Hence, it is not surprising that activation in the dorsal part of the ACC (dACC) is consistently reported in neuroimaging studies investigating decision processes in the UG (Sanfey et al., 2003; Gospic et al., 2011; Kirk et al., 2011); irrespective of participants' age (Guroglu et al., 2011). Further evidence for the involvement of the dACC in the context of the UG is provided by electrophysiological studies.

The medial frontal negativity (MFN), an event related potential which is supposed to be generated in the dACC (Gehring and Willoughby, 2002; Luu et al., 2003; Wessel et al., 2012), can be observed after the receipt of negative compared to positive feedback (Miltner et al., 1997; Luu et al., 2003; Nieuwenhuis et al., 2004b), after events that deviate from what we expect (Potts et al., 2006; Hajcak et al., 2007; Pfabigan et al., 2011), and in response to losses compared to gains (Gehring and Willoughby, 2002), irrespective of whether an action or choice preceded (Donkers et al., 2005; Martin et al., 2009). Furthermore, a similar negative deflection can be reported when we observe someone else receiving negative feedback or losing money (Fukushima and Hiraki, 2009). Generally, it is assumed that the MFN discriminates events on an abstract good-bad dimension (Nieuwenhuis et al., 2004a; Hajcak et al., 2006) or whether a goal has been achieved or not (Holroyd et al., 2006). Given that the MFN can be observed already 250 ms after the onset of an event, it serves as an index for early evaluation processes in economic decision making.

Having in mind that for some individuals the subjective value assigned to a certain reward highly depends on what others receive, the MFN should as well be modulated by social preferences like inequality aversion, altruism, or reciprocity. This has been confirmed in parts by studies investigating the UG. Fair offers elicited more positive MFN amplitudes than did unfair offers and are therefore preferred in view of the assumptions on the MFN (e.g., Boksem and De Cremer, 2010; Hewig et al., 2011). However, though results show that differences in MFN amplitude are related to concerns for fairness and rejection rate (Boksem and De Cremer, 2010; Hewig et al., 2011), it is unclear to what extent MFN amplitude differences between fair and unfair offers are affected by the proposer himself as a reference agent, or whether the MFN just differentiates between high and low amounts of money.

Findings of a recent study support the notion that the proposer accounts for alterations in the MFN. As outlined earlier one would expect a negative-going MFN after receiving an unfair offer. In their study they could show that social closeness between the proposer and the responder alters the polarity of the MFN amplitude. Offers made by a friend caused an inversion of the MFN (Campanha et al., 2011). However, a recent electrophysiological study investigated the influence of social comparison on behavior in the UG and MFN amplitudes by adding a social reference point, i.e., average proposals in other proposer-responder dyads were also presented to the responders (Wu et al., 2011); yet, no influence on the MFN amplitude could be reported. In a previous study, we added a human agent as a reference point by employing a three-person UG (Alexopoulos et al., 2012). This third player, a dummy-player so to speak, had no bearing in the game itself. Money had to be split up between all three players, and the responder, whose EEG was recorded during the game, had to accept or reject the allocation as otherwise customary in the standard UG. Results, as indicated by the MFN amplitudes, showed that responders only differentiated between fair and unfair offers toward themselves disregarding the share assigned to the dummy-player. However, offers that denoted a low share for the responder and a high share for the dummy-player elicited more pronounced MFN amplitudes than did offers with a low share for both players. This dissociation between the two kinds of unfair offers toward the responder might indicate that the third person had an impact on the responders' MFNs, and that he/she acts as the relevant reference agent responders care about. But though several studies suggested that empathic concerns are reflected in the MFN, the MFN observed in the responders seemed to be associated with negative social preferences. Nevertheless, it must be considered that participants were usually acquainted with each other whereas, in our study the dummy-players were unacquainted and in fact their presence was simulated. Therefore, one could assume that the actual presence of the dummy-player could have changed the direction of social preferences.

In the current study we therefore changed the setting and recorded EEG simultaneously from both recipients—the responder and the dummy-player—while they were playing the three-person UG using the same setting as reported in Alexopoulos et al. (2012). In doing so, we are able to clarify how the outcomes of the respective other are evaluated by participants who were treated fairly or unfairly themselves and to what extent agency influences concerns for fairness. Furthermore, we supposed that the actual presence of the third player changes the pattern of MFN amplitudes. Since several studies have shown that pre-play communication facilitates cooperation in social dilemma or bargaining games, respectively [for a survey see Crawford (1998)], we expected a similar effect on the early neural processes. More precisely, we expected a more negative MFN difference wave for unfair compared to fair offers assigned to the third player and an interaction of unfairness toward oneself with unfairness toward the other for unsubtracted, non-difference, ERP amplitudes. However the EEG of the dummy-player was recorded for two further reasons: First, we wanted them to be in the very same situation. Recording only the EEG of the responder could give rise to the feeling of being disadvantaged from the outset. Second, given that the dummy-players had no impact on the game, i.e., they could not punish unfair treatment, they acted as a yoked control group to clarify the impact of agency.

In addition to the ERP data individual concerns for fairness were collected, as previous studies reported that fairness concerns are related to MFN amplitude differences (Boksem and De Cremer, 2010). To this end we applied a justice sensitivity scale (Schmitt et al., 2004, 2010), which measures the degree to which individuals are concerned about injustice toward oneself and others.

## Materials and methods

### Participants

Thirty-six undergraduate students (16 males; mean age = 23.3 ± 2.69 years) from the University of Vienna participated in the experiment. All subjects were healthy, right handed, and naïve to the paradigm applied. Handedness was assessed using the Edinburgh Handedness Inventory (Oldfield, 1971). Subjects were paid between 15 and 20 Euros on average; actual earnings depended on their performance in the game.

The study was conducted in accordance with the *Declaration of Helsinki* (1973, revised in 1983) and local guidelines and regulations of the University of Vienna and the Faculty of Psychology. Written informed consent was obtained prior to the experiment.

### Justice sensitivity

Individual differences in the perception of justice were measured using the Justice Sensitivity Inventory (Schmitt et al., 2004, 2010). This 40-item questionnaire encompasses justice sensitivity from four different perspectives: the victim, the observer, the perpetrator, and the beneficiary. Each of the four subscales is covered by 10 questions that participants have to answer on a six-point Likert scale ranging from 0 to 5. Correlations between socially desirable and undesirable traits (Schmitt et al., 2004) as well as results from social bargaining games suggest that observer and beneficiary sensitivity reflect the degree to which a person is concerned about injustice toward others (Fetchenhauer and Huang, 2004). High scores on the domain victim sensitivity reflect concerns for justice toward oneself and are related to rather selfish behavior (Gollwitzer et al., 2009).

### Stimulus material

**Figure 1:**
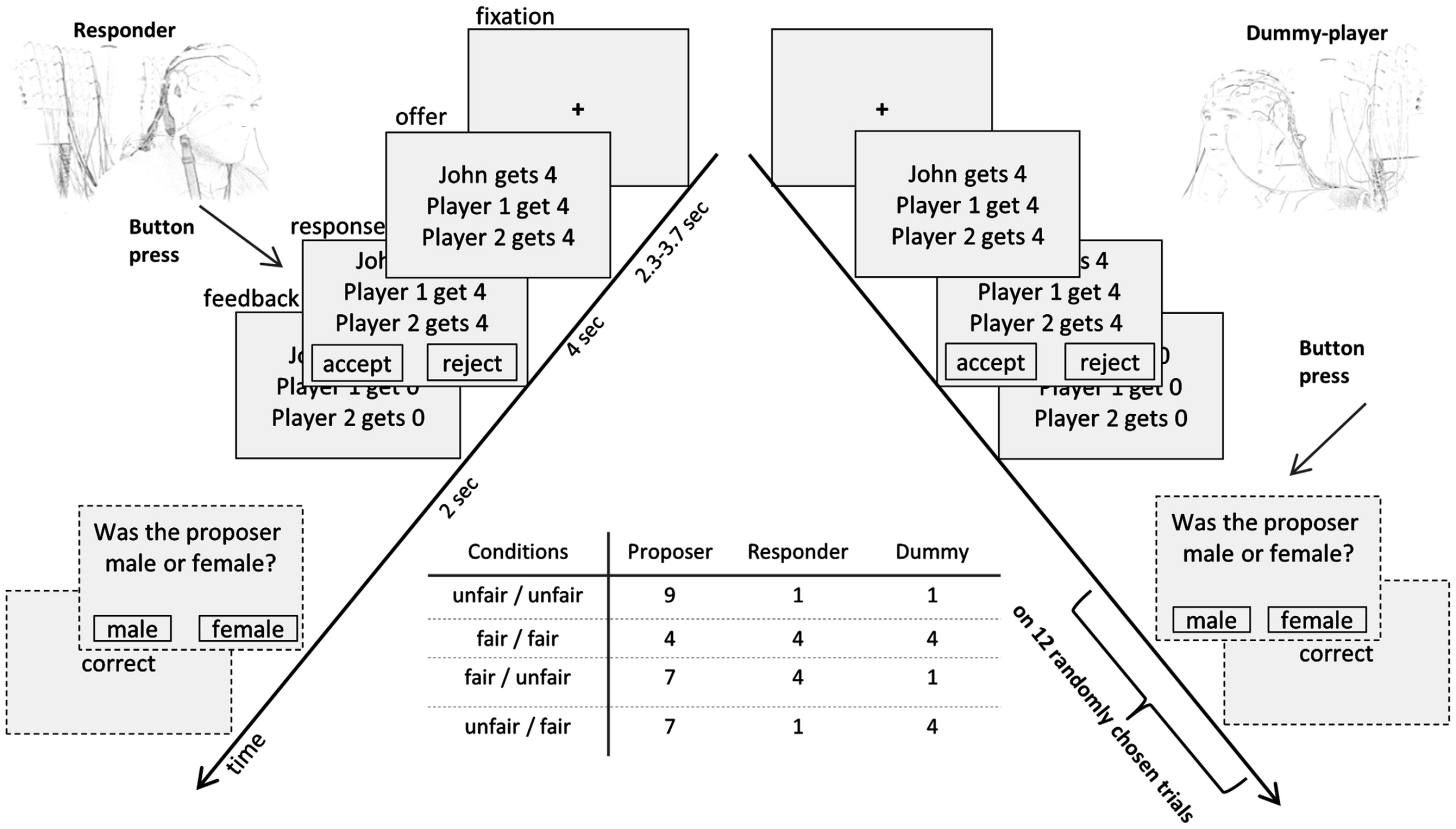
**Schematic representation of the three-person UG**. Structure of a single trial (for detailed description, see text) and the four conditions each with an exemplary allocation.

Altogether 324 proposals representing different divisions of the amount of 12, 15, or 18 Euros between the three players were presented. Half of these proposals were generated by the computer; the other half was provided by human proposers collected pre-experimentally [for details see Alexopoulos et al. (2012)]. In each of the two conditions (computer/human proposer) subjects received 27 fair offers (1/3 of the total amount for each player, hereinafter referred to as *fair/fair* offers) and 27 offers with an unfair share (less than 15%) for both receivers (referred to as *unfair/unfair* offers). 54 offers with an unfair share for one player only (receiving less than 15%, whereas the other one received 1/3), half of them with an unfair share for the responder (referred to as *unfair/fair*) and the other half with an unfair share for the dummy-player (referred to as *fair/unfair*). In addition, 54 offers were presented that did not meet any of the previous criteria and were therefore excluded from further analysis. In all conditions, the proposers allocated at least one-third of the total amount to themselves (see Figure 1 for examples of the different categories).

In accordance with our previous study (Alexopoulos et al., 2012) the presentation of these proposals, written in German (light gray background, black font color), consisted of three lines: the first line contained the amount the proposer (e.g., “John gets 4€”) or the computer (e.g., “The computer gets 4€”) wanted to keep, the second indicated the amount the responder, i.e., the participant, would receive (e.g., “Player 1 gets 4€”), and the third line indicated the amount the third player would get (e.g., “Player 2 gets 4€”). Offers were presented in six blocks with rest periods of varying duration in between. During these breaks both players were presented with the photographs of the proposers of the subsequent trials. Stimulus presentation was controlled by a Pentium IV 3.00 GHz computer using E-prime software (E-prime 2.0, Psychology Software Tools, Inc., Sharpsburg, Maryland).

### Paradigm and procedure

Participants were invited in gender-matched pairs. Upon arrival we ensured that these pairs were not acquainted with one another in any way. This was a precondition for the experiment to take place. Then they were informed about the further procedure, received written instructions concerning the nature of the three-person UG and were prepared for EEG recordings. Participants were allowed to introduce themselves to each other; however conversation was restricted to things unrelated to the experiment. In order to increase the feasibility of this setup and to emphasize that half of the proposals were made by human agents, both were shown the completed questionnaires of the proposers and were informed that they themselves, as well as the other players, would receive the amount of money they earned on four randomly chosen trials in their respective roles in this game. The roles (i.e., dummy-player or responder) were randomly assigned.

Throughout the experiment, the two sat opposite each other without eye contact in a sound-attenuated and dimly lit room. Both participants were seated in front of a 19-inch cathode ray tube monitor and were about 1.2 m apart from each other.

Each block of trials started with the introduction of the proposers, followed by 54 offers which had to be accepted or rejected by the subjects in the role of the responder (Figure 1). Trials were pseudo-randomized, hence each block contained the same number of human and computer offers. Offers were presented for 4000 ms followed by two squares apparent below the offer, each either containing the word “accept” or “reject.” These two alternatives changed their position randomly among the trials. Responders were instructed to press the corresponding button of a response pad (PST Serial Response Box by Psychology Software Tools, Inc.) with their right hand to indicate the chosen alternative. Subsequently feedback was given for the duration of 2000 ms. The format of the feedback was similar to the offer and indicated the actual allocation. Trials were separated by a variable inter-trial interval with a duration of 2300–2700 ms during which a black fixation cross was presented. At the end of each block, participants were informed about the amount of money they had gained so far followed by the introduction of the subsequent proposers. To maintain the attention of the other participant, i.e., the third player, 12 randomly chosen trials were followed by questions concerning the current offer (e.g.: Was the proposer male or female?). Below these questions two squares appeared, each of which either contained the word “yes” or “no” and subjects in the role of the third player had to press the corresponding button to answer. Subjects knew that for every correct answer both will receive 0.50 Euros additionally to the outcome of four randomly chosen trials.

### Electrophysiological recordings

EEG data from both subjects were recorded via 61 Ag/AgCl equidistantly located scalp electrodes embedded in an elastic cap (EASYCAP GmbH, Herrsching, Germany; montage M10), referenced to non-cephalic balanced sterno-vertebral electrodes (Stephenson and Gibbs, 1951). For eye movement artifact correction, vertical and horizontal electro-oculograms (VEOG, HEOG) were recorded bipolarly from above and below the left eye (VEOG), and from right and left outer canthi (HEOG). The subjects' skin was slightly scratched with a sterile needle at all recording sites in order to minimize skin potential artifacts and to ascertain homogeneous electrode impedances below 2 kΩ. Simultaneously recorded signals were amplified using two separate DC-amplifiers with high baseline stability and an input impedance of 100 GΩ (Ing. Kurt Zickler GmbH, Pfaffstätten, Austria). Signals were digitized with a 1 kHz sampling rate and recorded within a frequency range from DC (0 Hz) to 250 Hz. Synchronization of data collection was achieved using an external signal generator synchronizing the two DC-amplifiers.

### Data preprocessing

Eye movement and blink artifacts were first eliminated using a linear regression approach on the basis of parameters obtained in pre-experimental calibration trials (Bauer and Lauber, 1979). Using a template matching procedure blink coefficients were identified. Blink correction was then performed by subtracting vertical and horizontal EOG signals weighted this way from each EEG channel. Epochs of 1000 ms, 800 ms following stimulus (offer) onset and 200 ms preceding the onset, were extracted for the conditions fair/fair, unfair/unfair, fair/unfair, and unfair/fair (see Figure 1). For further data processing EEGLAB 6.03b was used (Delorme and Makeig, 2004). The 800 ms epochs were aligned to the 200 ms baseline preceding the presentation of the offer. Subsequently, data were down-sampled to 250 smp/s, low pass filtered (6 dB/octave slope) at 30 Hz cutoff, and linear trends were removed. To further improve data quality, e.g., correcting for artifacts occurring repeatedly, we followed the approach suggested by Delorme et al. (2007) which we already used and described in detail in Alexopoulos et al. (2012). According to Marco-Pallares and colleagues (2011), 10–20 trials are enough for measuring a reliable component, thus, subjects with less than 15 trials were excluded from further analysis. Thus, two pairs of subjects had to be excluded from further analysis since the remaining number of trials after artifact correction was too low. The remaining participants had on average 22.56 (*SD* = 2.2) trials per condition remained for each of the responders and 21.17 (2.3) for the dummy-players.

### Data analysis

Based on visual inspection of grand-averaged waveforms, scalp potential topography of difference waves, and in accordance with previous literature, the MFN was quantified as the average baseline corrected mean amplitude value in the time range between 220 and 320 ms after stimulus onset at electrode Fcz, Cz, and Pz (Alexopoulos et al., 2012; Boksem et al., 2012). Though statistical analyses revealed similar results for all electrodes; reported results are based on Cz since this electrode gave the highest effect sizes. Amplitude values of the MFN for the condition *human* and *computer* were submitted to 2 × 2 repeated measures ANOVAs with the factors *Self* (levels: fair and unfair share for oneself) and *Other* (levels: fair and unfair share for the other player) separately for both groups of subjects (responders and dummy-players). All factors were defined as within-subject factors.

Furthermore, to reduce confounding effects of other ERP components on the amplitude of the MFN and to scrutinize potential differences in processing of the outcome for the other recipient, we created difference waves. These difference waves were constructed by subtracting ERPs following fair offers from unfair offers toward the respective other, while the level of fairness toward oneself was kept constant. In this way we obtained two difference waves for each player: (1) *Self* fair, *Other* unfair minus fair, and (2) *Self* unfair, *Other* unfair minus fair. To test whether difference waves were statistically different from zero a one-sample *t*-test was applied.

To assess the relation between early neuronal processes and individual differences in justice sensitivity, MFN amplitudes, respectively the associated difference waves (unfair minus fair) at channel Cz were correlated with justice sensitivity scores (using Pearson correlation and two-tailed significance levels). Due to the low variability in acceptance rates we refrained from correlation analyzes of MFN amplitudes and decision behavior. For all analyses the significance threshold was set to *p* = 0.05. All statistical analyses were carried out with IBM SPSS Statistics 19 software (IBM Corp., Armonk, NY, USA).

## Results

### Performance

On average responders accepted 53% (*SD* = 43.15) of the offers made by the computer, compared to 52% (*SD* = 43.35) of offers made by human proposers. There was a statistically significant difference in acceptance rates depending on which type of offer was received, χ^2^(3) = 34.193, *P* = 0.000. *Post-hoc* analysis with Wilcoxon Signed-Rank Tests was conducted with Bonferroni correction applied, resulting in a significance level set at *p* < 0.008. Rejection rates were significantly higher for inequitable offers compared to equitable offers (for all comparisons *p* < 0.001). Offers with an unfair share for both players were rejected significantly more often than those which represented an unfair share to the dummy-player only (for details see Table 1).

**Table 1:** **Median (interquartile range) acceptance rates for human and computer proposers**.

	**Human**		**Computer**	
Fair (*R*)/Fair (*D*)	100%	(96–100)	100%	(96–100)
Unfair (*R*)/Unfair (*D*)	0%	(0–7)	2%	(0–7)
Unfair (*R*)/Fair (*D*)	33%	(0–75)	29%	(0–65)
Fair (*R*)/Unfair (*D*)	52%	(24–97)	67%	(24–97)

### ERP data

#### Responders

**Figure 2:**
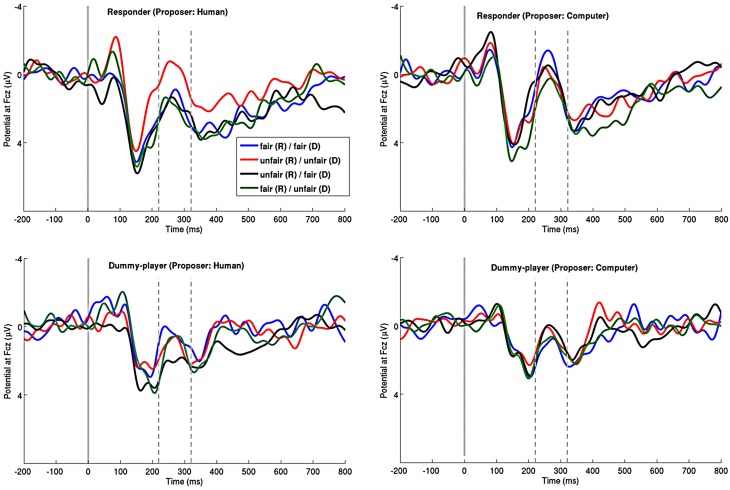
**Grand average ERP waveforms for each recipient and proposer at Cz for the offers: fair (*R*)/fair (*D*) (blue line), unfair (*R*)/unfair (*D*) (red line), unfair (*R*)/fair (*D*) (black line), or fair (*R*)/unfair (*D*) (green line); format: responder/dummy-player**. Negative is plotted up; Zeros on the timeline indicate the onset of the offer.

**Figure 3:**
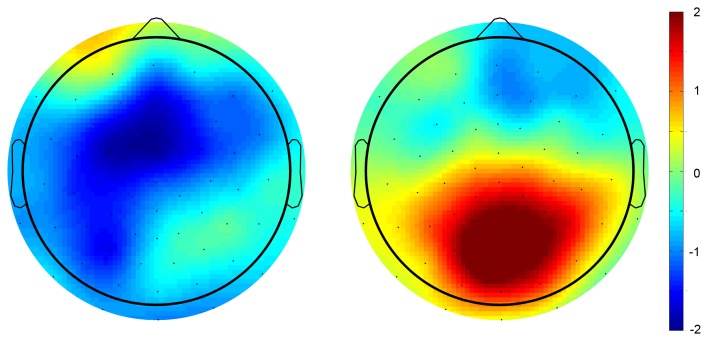
**Scalp potential topography of the average voltage differences between fair and unfair offers for the responder for the time point of the MFN (220–320 ms following offer onset)**.

**Figure 4:**
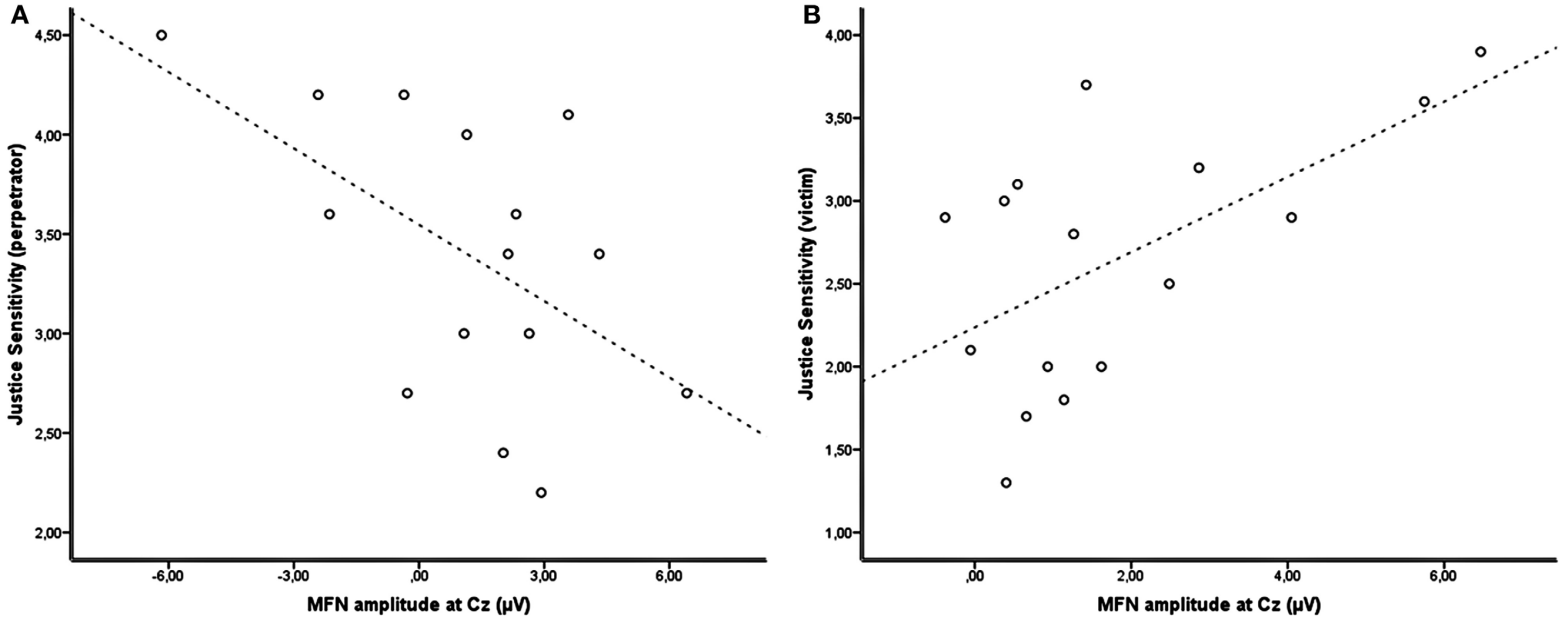
**Correlation between justice sensitivity scores and the difference in MFN amplitude between fair and unfair offers toward the respective other each with fair shares for oneself. (A)** MFN difference wave for fair and unfair offers toward the dummy-player [fair (*R*)/unfair (*D*) – fair (*R*)/fair (*D*)] and perpetrator sensitivity of the responders **(B)** MFN difference wave for fair and unfair offers toward the responder [unfair (*R*)/fair (*D*) – unfair (*R*)/fair (*D*)] and victim sensitivity of the dummy-player.

For the responders, mean MFN amplitudes in the time window 220–280 ms after a proposal made by a human agent revealed no significant main effect for the factor *Self*, [*F*_(1, 15)_ = 1.394, *p* = 0.256, partial η^2^ = 0.085] and the factor *Other* [*F*_(1, 15)_ = 1.396, *p* = 0.256, partial η^2^ = 0.085]. However, the interaction (*Self × Other*) was statistically significant [*F*_(1, 15)_ = 19.170, *p* = 0.001, partial η^2^ = 0.561]. Grand-average waveforms depicted in Figure 2 clearly show an increased MFN following offers with a low share for both recipients (unfair/unfair). Further analyses revealed that MFN amplitudes following this kind of offers were statistically significant compared to all other possible offers (for all *p* < 0.04). Likewise, only in cases where the responder received an unfair share, the amplitudes of difference waves (unfair/unfair minus unfair/fair) were significantly different from zero (mean = −2.352 μV, *t*_(15)_ = −4.452, *p* = 0.000) (see Figure 3). In case the responder received a fair share, however, no effect for high and low offers assigned to the dummy-player could be found (mean = 1.152 μV, *t*_(15)_ = 1.544, *p* = 0.144). The correlation analyzes of MFN difference waves and individual differences in justice sensitivity revealed a statistical relationship given by perpetrator sensitivity being negatively related to MFN amplitudes following proposals comprising unfair amounts toward the dummy-player (*r* = −0.553, *p* = 0.033). Thus, responders who are concerned about injustice toward others exhibit larger, more negative going, MFN amplitudes following advantageous inequality (see Figure 4 and Table 2).

**Table 2:** **Correlation between justice sensitivity and MFN difference wave**.

	**Responder**		**Dummy-player**	
	**Fair (*R*)/Unfair (*D*) – Fair (*R*)/Fair (D)**	**Unfair (*R*)/Unfair (*D*) – Unfair (*R*)/Fair (*D*)**	**Unfair (*R*)/Fair (*D*) – Fair (*R*)/Fair (*D*)**	**Unfair (*R*)/Unfair – Fair (*R*)/Unfair (*D*)**
Victim	−0.088	−0.259	−0.591^**^	−0.458^*^
Observer	−0.229	−0.048	−0.374	−0.033
Perpetrator	−0.553^*^	−0.001	−0.086	−0.036
Beneficiary	−0.356	−0.325	−0.211	−0.177

Note:

* p < 0.05,

** p < 0.01.

#### Dummy-player

Analysis of the mean MFN amplitudes for the dummy-players revealed a marginal non-significant interaction effect for *Self* × *Other*, [*F*_(1, 15)_ = 4.301, *p* = 0.056, partial η^2^ = 0.223]. The factor *Self* [*F*_(1, 15)_ = 0.001, *p* = 0.970, partial η^2^ = 0.000] and factor *Other* [*F*_(1, 15)_ = 3.507, *p* = 0.081, partial η^2^ = 0.189] again did not reach significance. Grand-averaged waveforms (see Figure 2) of the dummy-players indicate that compared to all other possible offers, those offers with a low share for only the responders (unfair/fair) are associated with a diminished negative going component. Consequently, only in case the dummy-player received a fair share, statistically significant differences between unfair and fair offers toward the responder could be observed [mean = 1.846, *t*_(15)_ = 3.672, *p* = 0.002]. In case the dummy-player received an unfair amount, no difference in MFN amplitudes associated with unfair compared to fair offer toward the responder could be observed [mean = −0.091 μV, *t*_(15)_ = −0.116, *p* = 0.909]. The relation between justice sensitivity and MFN difference wave was analyzed similar to the responders' data. Victim sensitivity was positively related to MFN difference waves following offers with an unfair share for the responder, regardless of whether the dummy-player received a fair share (*r* = 0.591, *p* = 0.008) or an unfair share (*r* = 0.458, *p* = 0.037; see Figure 4 and Table 2). Accordingly, dummy-players who were more concerned about injustice toward themselves exhibited larger positive going MFN amplitudes following unfair offers for the responder.

None of the statistical analyses applied to the ERP data associated with proposals made by the computer reached significance—neither for the responders (*p* > 0.242) nor for the dummy—players (*p* > 0.328). Furthermore, we found no differences between the responders and the dummy-players with regard to justice sensitivity (*p* > 0.296 for all four scales).

## Discussion

In the current study in contrast to previous studies two participants were recorded simultaneously while playing the three-person UG. Both participants played the part of the receivers with one of them in the role of the dummy-player having no say. The responder, on the other side, had veto power and thus, was able to harm the payoff of all other players. These differences in power became apparent already about 250 ms after the onset of the different offers. For both participants a difference in MFN amplitude depending on the share assigned to the respective other can be reported. In line with previous literature, MFN amplitudes elicited by unfair offers were more negative going than those elicited by fair offers, but this only applied for the responders. The dummy-players showed to some extend the opposite pattern; unfair offers compared to fair offers toward the responder were followed by positive-going amplitudes within the time range of the MFN. Although, we found differences between MFN amplitudes when the offer is made by a human proposer, no difference in MFN amplitudes could be observed following proposals made by the computer, neither for the responder nor for the dummy-player. This might be surprising at first since acceptance rates did not differ substantially between these two conditions. However, considering that we have to differentiate between at least two different processes this might become more comprehensible. The MFN is associated with the subjective value assigned to a certain situation (Holroyd and Coles, 2008; Rigoni et al., 2010); whereas, value is derived by comparative processes. Thus, expectations or prior experience and available options change the absolute value of a given reward and the associated MFN amplitude. Several studies have shown that social processes are also reflected in the amplitude of the MFN [for a review see Thoma and Bellebaum (2012)], since experiences in social interactions drive the expectations we have regarding the behavior of other individuals. Therefore, it might not be too surprising that no substantial differences in the MFN amplitude can be observed between conditions when the computer acts as a proposer, especially since offers are randomized and, regarding the offer size, evenly distributed. This might suggest that the intentions of the proposers indeed influence the initial evaluation process, however do not necessarily determine whether an unequal offer is accepted or not. After all it is still not a pure computer condition, since the dummy-player still has to be considered in the current decision process. Similar results were obtained in a study in which a random number generator decided how to split the money between two players. This study was also able to show that the ACC and the medial prefrontal cortex, both regions that have been associated with the MFN component especially in the context of the UG (Campanha et al., 2011; Billeke et al., 2012), are involved in the processing of unequal offers only when the participants themselves were affected. Moreover, no activation increase could be observed in this cluster when decisions were made for someone else without the participant being directly affected, although unequal offers were still rejected (Civai et al., 2012).

Regarding the results of responders; a recent attempt to investigate MFN amplitude changes in the context of the three-person UG found that responders did not differentiate between fair and unfair offers assigned to the dummy-player (Alexopoulos et al., 2012). Nevertheless, offers that clearly favored the dummy-player opposed to the subjects themselves were followed by the most pronounced MFN amplitudes. In contrast, offers that were equally unfair for both—the dummy-player and the responder—did not reveal distinct MFN amplitudes. Being speculative, anger toward the proposer and envy toward the dummy-player may have led to the increase in amplitude. In contrast to the present study these two recipients were anonymous to each other. We assume that the change in experimental setup has led to the observed differences in the ERP patterns of the responders. In the present study offers with an equally low share for the two recipients elicited the most pronounced, negative going, amplitude at the time a MFN is usually observed. This suggests that offers comprising a fair share for at least one of the two recipients are evaluated nearly as satisfying as offers with an equally high share for all three players. Furthermore, responders clearly differentiated between high and low offers assigned to the dummy-player, with low offers leading to a more negative going MFN, at least when they themselves received an unfair share as well.

It is well known that pre-play communication enforces cooperation in social dilemma games or bargaining games, respectively [for a survey see Crawford (1998) or Greiner et al. (2005)] investigating pre-play communication in the three-person UG. In line with this finding there are at least two explanations for the changes in MFN amplitudes: Strategic issues, since the reputation of the responder is at risk, or changes in utility, since group identity enhances “we” feelings among group members, commonly summarized as emphatic concerns (Greiner et al., 2010). Recent efforts in the field of social neuroscience provide evidence that empathy is modulated by perceived group membership (Hein et al., 2010) and that empathy-related processes are expressed in the appearance of the MFN. Receiving negative feedback is associated with an increase in MFN amplitude. Observing someone else receiving negative feedback similarly elicits a MFN. Whereas, the magnitude depends on the perceived similarity with the other (Carp et al., 2009), the closeness (Kang et al., 2010), self-reported levels of empathy (Fukushima and Hiraki, 2009), and the degree to which participants include others in their self-concept (Kang et al., 2010). Since the MFN is supposed to be generated in the ACC, the fact that the ACC is a key structure implicated in the empathic response to physical and social pain of others (Singer et al., 2004; Masten et al., 2011), further suggests that empathic concerns over strategic issues have influenced the appearance of the MFN. This view is further supported by the relation between justice sensitivity and MFN amplitudes found in the present study.

Even though MFN amplitudes did not differentiate between high and low offers assigned to the dummy-player in cases were responders received a high share, the mean amplitude of MFN difference waves varied with the degree to which subjects reported to be concerned about injustice toward others. Boksem and De Cremer (2010) already reported that MFN amplitudes following unfair offers in the standard UG varied with self-reported concerns for fairness and honesty.

In the present study the degree to which responders included the share for the dummy-player when they themselves received a fair share in the evaluation process, similarly varied with their concerns for fairness. Responders scoring high on perpetrator sensitivity exhibited larger MFN amplitudes following advantageously unequal offers. Perpetrator sensitivity is highly related to socially desirable traits as well as to cooperative behavior in social dilemma games (Schmitt et al., 2004; Gollwitzer et al., 2005). Since perpetrator sensitivity focuses on situations where people actively take advantage of another party, it is assumed to be linked to feelings of guilt (Thomas et al., 2010). For instance, one example for perpetrator sensitivity would be “I feel guilty when I am better off than others for no reason.” Hence, this kind of discomfort might be reflected in higher, more negative-going, MFN amplitudes in response to unfair offers toward the dummy-player. In other words, feelings of guilt might reduce the value of the relatively high share assigned to the responder in the light of a low, unfair share toward the dummy-players.

Regarding the results of the dummy-players; also MFN amplitudes of the participants playing in the role of the dummy-player were related to justice sensitivity, though, a somewhat different picture is emerging. First of all, the dummies' MFN amplitudes, though differing with respect to the outcome of the responder, were more pronounced for fair than unfair offers toward the responder. This is in contrast to what one would expect considering the data of the responders. Nevertheless, Marco-Pallares and colleagues (2010) showed that in a competitive setting observing someone else receiving a gain led to higher, more negative-going MFN amplitudes, whereas in neutral conditions MFN amplitudes were higher following losses as compared to gains of the performer. Second, offers with low shares for the responder and high shares for the dummy-player elicited a MFN difference wave significantly different from zero, but again with positive polarity. Furthermore, the higher the scores of the dummy-players were on the victim sensitivity scale, the more positive amplitudes following low offers for the responders could be observed. Victim sensitivity covers situations associated with injustice toward oneself and is related to socially undesirable traits like vengeance, jealousy and distrust. In bargaining games victim sensitive individuals tend to be less cooperative, i.e., they offer less in the UG or dictator game (Gollwitzer et al., 2005). The dummy-players are at a disadvantage from the outset, because they have no influence on the proposed allocation. This might have led to the finding that advantageous, unequal offers are more favorable than any other possible offer and even more so in subjects who are generally more concerned about fairness toward themselves. In contrast to our previous study where the responders reacted merely selfish, the presence of the dummy-player seemed to enforce “we” feelings and empathic concerns. However, this time likewise the responders in the anonymous setting, the powerless players experienced negative social preferences. While there are parallels between those two, there are also substantial differences: Responders in the anonymous setting preferred all other offers over those that assigned a low share to themselves and a high share toward the other. Therefore, we assume that envy might play a crucial role. In contrast, the powerless players preferred offers with low shares toward the responder and high shares toward themselves, which might be more closely related to spite.

However, there is another possible explanation regarding the MFN of the dummy-players: It is assumed that the MFN distinguishes events on an abstract good-bad dimension (Nieuwenhuis et al., 2004a) or in other words indicates whether a goal has been achieved or not (Hajcak et al., 2006). This is achieved by taking into account prior knowledge or available alternatives to adapt to a changing environment and facilitate future behavior. Positive and negative reward prediction errors determine the amplitude of the MFN, unpredicted positive events decrease the amplitude and negative events increase the amplitude. In light of the assumptions concerning the appearance of the MFN this might suggest that in the social context rather the expectations regarding other people's behavior and not merely reward and punishment itself influence the amplitude of the MFN. Thus, one can argue that dummy-players might have anticipated receiving lower offers than the responders, therefore high offers for the dummy-player were an unexpected reward leading to a reduction in MFN amplitude.

To conclude, in the present study we showed that the influence of agency and physical distance on social preferences can already be observed at an early level of neural processing. As participants were unfamiliar to each other prior to the experiment and we did not control for sympathy, future research has to show how the level of familiarity or sympathy will further enforce this “we” feelings.

### Conflict of interest statement

The authors declare that the research was conducted in the absence of any commercial or financial relationships that could be construed as a potential conflict of interest.
